# Occupational Exposures to Organic Dust in Irish Bakeries and a Pizzeria Restaurant

**DOI:** 10.3390/microorganisms8010118

**Published:** 2020-01-15

**Authors:** Carla Viegas, Gerard T. A. Fleming, Abdul Kadir, Beatriz Almeida, Liliana Aranha Caetano, Anita Quintal Gomes, Magdalena Twarużek, Robert Kosicki, Susana Viegas, Ann Marie Coggins

**Affiliations:** 1 H&TRC-Health & Technology Research Center, ESTeSL-Escola Superior de Tecnologia da Saúde, Instituto Politécnico de Lisboa, 1990-096 Lisbon, Portugal; beatrizltalmeida1@gmail.com (B.A.); liliana.caetano@estesl.ipl.pt (L.A.C.); anita.gomes@estesl.ipl.pt (A.Q.G.);; 2 NOVA National School of Public Health, Public Health Research Centre, Universidade NOVA de Lisboa, 1600-560 Lisbon, Portugal; 3 Comprehensive Health Research Center (CHRC), 1150-090 Lisbon, Portugal; 4 School of Natural Sciences and Ryan Institute, National University of Ireland, H91 CF50 Galway, Ireland; ger.fleming@nuigalway.ie; 5 School of Physics and Ryan Institute, National University of Ireland, H91 CF50 Galway, Ireland; abdulkadirindo22@gmail.com; 6 Research Institute for Medicines (iMed.ULisboa), Faculty of Pharmacy, University of Lisbon, 1649-003 Lisbon, Portugal; 7 Institute of Molecular Medicine, Faculty of Medicine, University of Lisbon, 1649-028 Lisbon, Portugal; 8 Department of Physiology and Toxicology, Faculty of Biological Sciences, Kazimierz Wielki University, Chodkiewicza 30, 85–064 Bydgoszcz, Poland; twarmag@ukw.edu.pl (M.T.); robkos@ukw.edu.pl (R.K.)

**Keywords:** occupational exposure assessment, microbial contamination, *Aspergillus*, azole resistance screening, mycotoxins

## Abstract

For decades, occupational exposure to flour dust has been linked to a range of respiratory diseases, including occupational asthma, thought to result from exposure to fungi present in the flour. Antifungal resistance is of increasing prevalence in clinical settings, and the role of occupational and environmental exposures, particularly for specific fungal species, is of concern. Occupational exposure to flour dust can occur in a range of occupational settings, however, few studies have focused on restaurant workers. The objective of this study was to measure occupational exposure to flour and microbial contamination, including azole resistance screening, in two small commercial bakeries and in a pizzeria. Personal full shift inhalable dust measurements were collected from workers, and were analyzed for inhalable dust and fungi, bacteria, azole resistance, and mycotoxins. Samples of settled dust were collected, and electrostatic dust cloths (EDC) were deployed and analyzed for microbial contamination, including azole resistance screening, and mycotoxins. Geometric mean exposures of 6.5 mg m^−3^ were calculated for inhalable dust, however, exposures of up to 18.30 mg m^−3^ were measured—70% of personal exposure measurements exceeded the occupational exposure limit for flour dust of 1.0 mg m^−3^. The air and EDC fungal counts were similar to those reported in previous studies for similar occupational environments. The fungi were dominated by *Penicillium* genera, however *Aspergillus* genera, including *Fumigati* and *Flavi* sections, were observed using culture-based methods, and the *Fumigati* section was also observed by molecular tools. Both *Aspergillus* sections were identified on the azole resistance screening. Mycotoxins were also detected in the settled dust samples, dominated by deoxynivalenol (DON). The role of environmental exposure in both the development of antimicrobial resistance and the total mycotoxin body burden is a growing concern; therefore, the presence of azole-resistant fungi and mycotoxin contamination, although low in magnitude, is of concern and warrants further investigation.

## 1. Introduction

Flour, the basic ingredient in an array of bread and bakery products, is a complex organic dust containing allergens and antigenic particles from constituent cereals, such as wheat, oat, rye, rice, or corn [1]. Along with other raw ingredients commonly used in baking, baker’s yeast, bread improvers, and flour can provide an ideal substrate for microbial growth, and can generate high levels of bioaerosols during processing [2,3]. Occupational exposures in this sector can lead to the development of conjunctivitis; contact dermatitis; and debilitating occupational respiratory diseases, including flour induced rhinitis and “baker’s asthma”, the latter of which is one of the most common work-related respiratory diseases. Wheat sensitization prevalence rates of up to 30% have been reported for bakers [4,5,6,7,8], along with increased rates of childhood asthma among bakers’ children, believed to be as a result of parental occupational exposures to flour dust in bakers’ homes [9]. Baker’s asthma may occur as a result of immunological sensitization following exposure to wheat allergens, in particular *Aspergillus* derived α-amylase or trypsin, which are often present in flour dust [10,11]. 

An increased prevalence of respiratory and asthmatic symptoms has been reported at dust exposures of 1.5–4.0 mg m^−3^, with sensitization to flour dust being reported after exposures as low as 0.5 mg m^−3^ [6]. Additionally, occupational exposure to azole-resistant strains of fungi, such as *Aspergillus* sp., and toxigenic substances, such as mycotoxins, have been detected in cereals such as wheat, rye, oats, and corn, and are also of concern in this sector [2], especially with reports of the increasing prevalence of azole-resistant strains of fungal species in clinical settings [12].

Despite the clear association between exposure to flour dust and adverse health outcomes, there is no clear downward trend in exposure within this sector [13,14]. Recent United Kingdom statistics suggest that over the period of 2017–2018, the second highest rate of occupational asthma (40.0 per 100,000) was among bakers and confectioners [15]. Similarly, high rates of baker’s asthma have previously been reported in France [16], Norway [17], Finland [18], and Poland [19]. In Ireland, asthma diagnoses comprise the largest proportion of cases of occupational respiratory disease (36%) reported to ROI-SWORD over the period of 2005–2016. After isocyanates, flour dust is one of the agents associated with the 59 diagnoses of occupational asthma, and is the most frequently reported agent in Northern Ireland [20]. 

Exposure to flour dust and allergens can occur in a wide variety of occupational settings, from grains mills, animal feed plants, bakeries (bread and confectionary), supermarket bakeries, pasta factories, pizzerias, and restaurants [21]. The size of the bakery, the job, or work task performed are important determinants of exposure [22,23], with higher exposures reported for tasks involving sieving flour and other dry ingredients [23], kneading of dough [21], baking [24], or cleaning operations [25]. Additionally, the presence or use of engineering controls within bakeries tends to be poor [22,23,24].

In recent years, there has been an increasing trend in the Irish bread products market [26], it is estimated that there are currently 550 active enterprises engaged in the manufacture of bread, pastry, and pasta in Ireland, employing over 7000 workers [27]. This number does not include those working in pizzerias or hotel bakeries. This study aimed to assess personal exposure to flour, fungi, and bacteria (bioburden), including azole resistance screening and mycotoxins in two small commercial bakeries and one pizzeria in Ireland.

## 2. Materials and Methods

### 2.1. Study Participants 

One pizzeria and two commercial bakeries owners agreed to participate on the study. They were located in the west of Ireland and surveyed over the period of June and July 2018. The pizzeria restaurant and Bakery 1 employed one worker each who performed all of the work tasks. Bakery 2 had two workers who performed similar work tasks. The pizzeria restaurant consisted of two work areas, one area included the raw ingredients store, where materials were added to a kneading machine to produce the pizza or bread dough, which was then transferred to a second area where it was kneaded by hand and used to prepare pizzas, breads or pastries. In Bakery 2, similar tasks to those described in the pizzeria restaurant were performed. In Bakery 1, tasks were performed across two rooms, depending on the products produced (bread or pastry). During the surveys, contextual information regarding the type of flour used, work tasks performed by the workers, exposure controls available, number of bakeries products produced, and ingredients used during the production were recorded, and are presented in Table 1.

### 2.2. Flour Dust Exposure Assessment 

**Figure 1:**
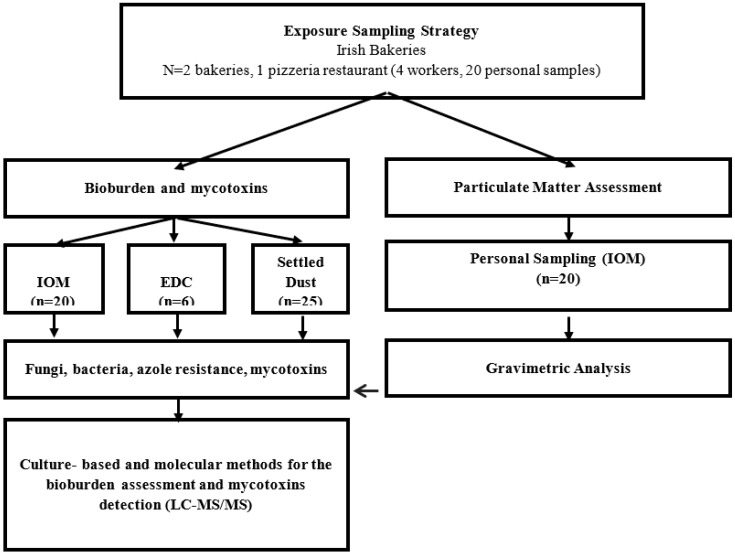
Sampling approach used for the occupational exposure assessment. EDC—electrostatic dust cloths.

The objective was to collect full shift (8 h) personal samples to assess flour dust exposure, which typically included the mixing of raw materials, hand kneading, and baking. Personal exposure measurements were collected and analyzed following HSE MDHS 14/4 [28]. The samples were collected in the worker breathing zone using portable SKC Sidekick sampling pumps connected to an IOM sampler SKC, Ltd., Dorset, UK containing 25 mm Whatman GF/A glass microfiber filters (pre-sterilized by autoclaving at a standard temperature and pressure; Figure 1). The pumps operated at 2.0 L m^−1^, and were pre- and post-calibrated using a DryCal^®^ DC Lite primary calibrator (BIOS International, Pompton Plains, NJ, USA). Workers wore the sampling train for the full sampling period, which varied from 3–8 h. The sampling period did not include worker break periods (40–45 min), and the sample duration was based on the availability of the workers performing the work tasks. Sample filters were handled aseptically and analyzed for inhalable dust gravimetrically using a Sartorius ME5 microbalance (precision 20 µg), and then prepared for the microbial analysis. After gravimetric analysis, each filter was extracted in sterile 10 mL deionized water with 0.05% Tween80™ (Sigma-Aldrich, Dorset, UK) at 250 rpm for 1 min, and then 3.8 mL of sterile glycerol was added and the solution was extracted again for 1 min at 250 rpm, then stored at −80 °C until the microbial analysis. 

### 2.3. Environmental Samples

Samples of settled dust were collected and electrostatic dust cloths (EDCs; surface area of 0.02 m^2^) were deployed for 15 days (passive sampling methods are shown in Figure 1). The settled dust and EDC samples were used to estimate the long-term exposure and facilitate a more detailed analysis of the microbial burden, including mycotoxins. Approximately 5 g of settled dust was collected using sterilized stainless-steel spatulas into pre-sterilized bags. Immediately after sampling, 4.4 g of the dust was extracted with 40 mL of distilled water for 20 min at 200 rpm, as previously described [29,30,31,32].

The EDCs were placed in sterilized petri dishes at a minimum height of approximately 0.93 m above floor level. After sampling, each EDC was extracted with 20 mL 0.9% NaCl with 0.05% Tween80™ by orbital shaking (250 rpm, 60 min, at room temperature) [29]. All of the sample extracts were stored at −80 °C, with glycerol added and analyzed four weeks after collection.

#### 2.3.1. Characteristics of Bacterial Contamination

The sample bacteria loading was quantified after serial dilution in sterile PBS and spread-plating to tryptic soy agar (TSA, Frilabo, Maia, Portugal) supplemented with 0.2% nystatin and violet red bile agar (VRBA, Frilabo, Maia, Portugal), and the samples were incubated at 30 °C and 35 °C for 7 days, respectively. The bacteria densities (colony-forming units: CFU·m^−3^, CFU·m^−2^ and CFU·g^−1^) were determined on the different culture media.

#### 2.3.2. Fungal Characterization and Azole Resistance Screening Using Culture-Based Methods

The fungal contamination was determined through the inoculation of 150 µL of the wash suspension from the collected samples on 2% malt extract agar (MEA, Frilabo, Maia, Portugal), supplemented with chloramphenicol (0.05%) and dichloran glycerol (DG18, Frilabo, Maia, Portugal). The prevalence of azole-resistance was determined in IOM filters, settled dust, and EDC samples using azole-supplemented media by seeding 150 µL of the wash suspension on Sabouraud dextrose agar (SDA) supplemented with 4 mg/L itraconazole (ITRA), 1 mg/L voriconazole (VORI), or 0.5 mg/L posaconazole (POSA), adapted from the EUCAST guidelines [33,34]. All of the collected samples were incubated at 27 °C for 5–7 days, in order to allow for the growth of all of the fungal species present in the samples. 

After incubation, quantitative (colony-forming units: CFU·m^−3^, CFU·m^−2^, and CFU·g^−1^) and qualitative results were obtained, and isolated fungal genera or species/sections were identified. Microscopic mounts were performed using a tease mount or Scotch tape mount and lactophenol cotton blue mount procedures, and the morphological identification from all of the fungi was performed using macro- and micro-scopic characteristics, as reported previously [35].

#### 2.3.3. Fungal Detection Using Molecular Tools

The molecular identification of the different *Aspergillus* species (*Circumdati, Flavi, Fumigati,* and *Versicolores*) was performed using real-time PCR (qPCR) using the Via 7 Real-time PCR System (Applied Biosystems, Forster City, CA, USA) on settled dust and EDC samples (*n* = 51), following previously published procedures, and using primers and probes [36]. For each gene that was amplified, a non-template control and a positive control consisting of DNA obtained from a reference that belonged to the culture collection of the Reference Unit for Parasitic and Fungal Infections, Department of Infectious Diseases of the National Institute of Health, from Dr. Ricardo Jorge. These strains have been sequenced for ITS B-tubulin, and Calmodulin.

#### 2.3.4. Mycotoxins Analysis

Twenty-five samples (5 from Bakery 1 and 10 each from Bakery 2 and the pizzeria restaurant) of settled dust were screened for the presence of mycotoxins. Settled dust samples (0.25 g) were extracted with 1.0 mL of ACN:H_2_O:AcOH (79:20:1) for 60 min. Raw extracts were diluted with the same amount of water, and were mixed, filtered, and injected into the LC-MS/MS system (Shimadzu, Tokyo, Japan). Similar methodologies for the detection of mycotoxins were followed to those reported in previous studies [2,3]. Several mycotoxins were targeted in the assessment performed, namely: patulin (PAT), nivalenol (NIV), deoxynivalenol-3-glucoside (DON-3-G), deoxynivalenol (DON), fusarenon-X (FUS-X), α-zearalanol (α-ZAL), β-zearalanol (β-ZAL), α-zearalenol (α-ZEL), zearalanone (ZAN), zearalenone (ZEN), T2 tetraol, deepoxydeoxynivalenol (DOM-1), neosolaniol (NEO), 15-acetyldeoxynivalenol (15-AcDON), 3-acetyldeoxynivalenol (3-AcDON), monoacetoxyscirpenol (MAS), diacetoxyscirpenol (DAS), aflatoxin M1 (AFM1), aflatoxin B1 (AFB1), aflatoxin B2 (AFB2), aflatoxin G1 (AFG1), aflatoxin G2 (AFBG2), fumonisin B1 (FB1), fumonisin B2 (FB2), fumonisin B3 (FB3), T2 triol, roquefortine C (ROQ-C), griseofulvin (GRIS), T2 toxin, HT2 toxin, ochratoxin A (OTA), ochratoxin B (OTB), mycophenolic acid (MPA), mevinolin (MEV), sterigmatocystin (STER), and indomethacin (IDN). The limits of detection (LOD) and quantification (LOQ) for each mycotoxin are presented in Table 2.

### 2.4. Statistical Analysis

The data were analyzed using SPSS statistical software, v22.0 for Windows (Microsoft, Lisbon, Portugal). The results were considered significant at a 5% significance level. To test the normality of the data, the Shapiro–Wilk test was used. The concentration data were not normally distributed, and thus Spearman’s correlation was used to study the relationship between the flour dust concentrations, and fungal and bacterial bioburden, and a Kruskal–Wallis test was used to compare the fungal concentrations in MEA, TSA, and Gram-negative media for settled dust, personal samples, and EDC. 

## 3. Results

**Figure 2:**
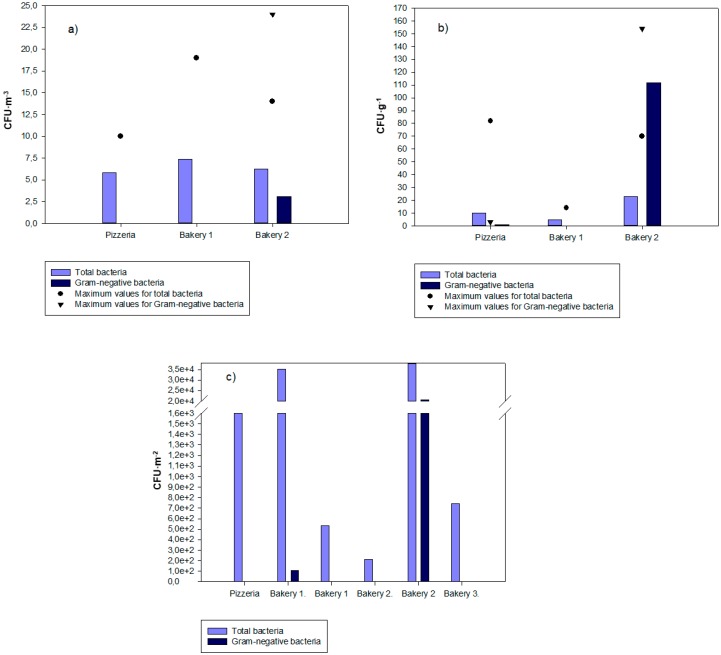
Bacterial contamination in (**a**) the IOM filter samples (average), (**b**) settled dust samples (average), and (**c**) EDC samples.

**Figure 3:**
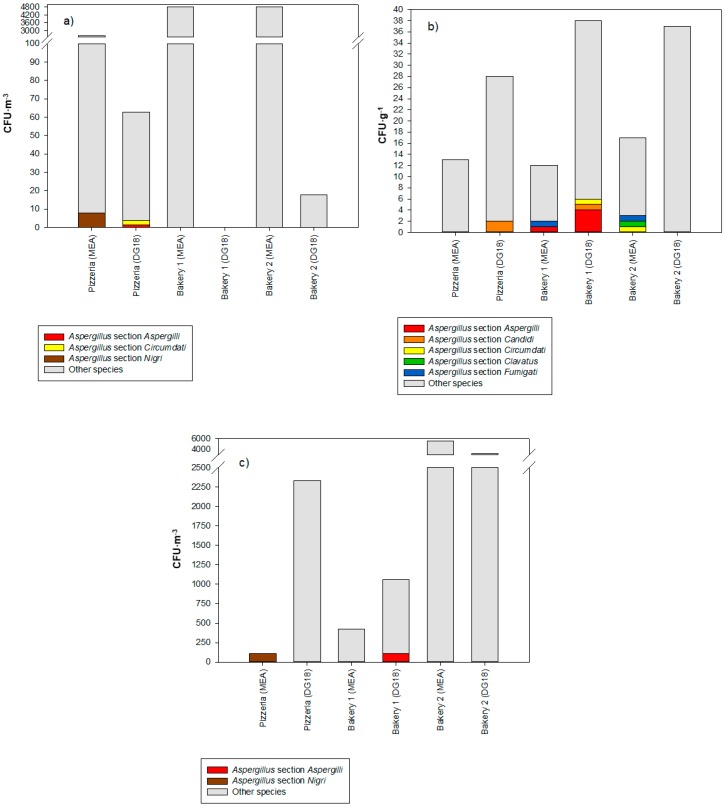
*Aspergillus* sections found in (**a**) IOM filter samples, (**b**) settled dust samples, and (**c**) EDC samples.

A total of 20 personal exposure measurements (5 samples in the pizzeria restaurant and 15 in the participating bakeries) were collected and analyzed for the total inhalable dust, fungi (including azole-resistant fungi), and bacteria. The sampling times ranged from 185–385 min. A total of 25 samples of settled dust were collected and analyzed for mycotoxins and fungi, and a total of six EDCs (two per participating workplace) were also analyzed for fungi. The results are presented in Table 3, Table 4, Table 5 and Table 6 and Figure 2 and Figure 3. There were no exposure controls provided in either bakery, and the workers did not use respiratory protective equipment. 

### 3.1. Personal Flour Dust Exposure Levels: Total Inhalable Dust

Higher flour dust concentrations were recorded in the bakeries compared with the pizzeria restaurant. Although similar work activities were performed in both bakeries, Bakery 2 had significantly higher (*p* < 0.05) flour dust exposures than Bakery 1 (Geometric mean (GM); 10.14 mg·m^−3^ compared with 2.66 mg·m^−3^ in bakery 1). Inhalable flour dust exposures, expressed as 8 h time weighted average’s (TWAs) ranged from 0.50 to 8.40 mg·m^−3^, 70% exceeded the Occupational Exposure Limit Value (OELV) for flour dust of 1.0 mg·m^−3^. The flour dust concentrations were positively correlated with the total amount of flour used on the day of sampling (*p* < 0.05; Table 3).

### 3.2. Bacterial Contamination Distribution

The bacterial contamination ranged from 0 CFU·m^−3^ to 19 CFU·m^−3^ (Bakery 1) on the IOM filter samples, 1 CFU·g^−1^ to 82 CFU·g^−1^ (pizzeria restaurant) in the settled dust samples, and from 212 CFU·m^−2^ (in Bakery 1) to uncountable (in Bakery 2) on the EDC (Figure 2).

Gram-negative bacterial contamination ranged from 0 CFU·m^−2^ to 24 CFU·m^−3^ (Bakery 2) on the IOM filter samples (Table 4), 0 CFU·g^−1^ to uncountable in Bakery 2 in the settled dust samples, and from 0 CFU·m^−2^ to 20.5 × 10.5 × 10^3^ CFU·m^−2^ (in Bakery 2) in the EDC samples (Figure 2). 

### 3.3. Fungal Contamination Characterization

Personal exposure to fungi (IOM filter samples) ranged from 0 CFU·m^−3^ to 1331 CFU·m^−3^ (Bakery 1) on MEA, and from 0 CFU·m^−2^ to 34 CFU·m^−2^ (pizzeria restaurant) on DG18 (Table 4). The fungal contamination in the settled dust samples ranged from 0 CFU·g^−1^ to 5 CFU·g^−1^ (pizzeria restaurant and Bakery 1) on MEA, and from 0 CFU·g^−1^ to 17 CFU·g^−1^ (Bakeries 1 and 2) on DG18. The fungal contamination on the EDC samples ranged from 0 CFU·m^−2^ to 3.3 × 10.3 × 10^3^ CFU·m^−2^ (Bakery 2) on MEA, and from 106 CFU·m^−2^ (Bakery 1) to 3.3 × 10^3^ CFU·m^−2^ (Bakery 2) on DG18.

Overall, the most common species, identified in the personal exposure (on MEA), were *Chrysonilia sitophila* (99.53%), *Penicillium* sp. (0.36%), and *Aspergillus* section *Nigri* (0.06%); and in DG18, were *Penicillium* sp. (91.08%), *Aspergillus* sp. (4.80%), and *Cladosporium* sp. (4.12%). Table 5 presents the fungal distribution by the units assessed.

The most prevalent fungi observed in the settled dust samples were *Penicillium* sp. (81.25%), *Aspergillus* sp. (15.63%), and *Mucor* sp. (3.13%) on MEA; and *Penicillium* sp. (88.35%), *Aspergillus* sp. (7.77%), and *Chrysosporium* sp. (2.91%) on DG18. Table 6 shows the fungal distribution by units assessed.

Finally, with regard to the EDC samples, the most prevalent species identified were *Penicillium* sp. (86.21%), *Chrysosporium* sp. (8.62%), *Mucor* sp. (1.72%), *Aspergillus* section *Nigri* (1.72%) and *Mucor* sp. (1.72%) on MEA, and *Penicillium* sp. (80.95%), *Cladosporium* sp. (14.29%) and *Aspergillus* sp. (1.59%), *Monascus ruber* (1.59%), and *Mucor* sp. (1.59%) on DG18. Table 7 present the fungal distribution by units assessed.

Different *Aspergillus* sections were detected depending of the sampling method used. However, a more diverse *Aspergillus* burden was detected in the settled dust samples (Figure 3).

### 3.4. Fungal Load in Azole-Supplemented Media

**Figure 4:**
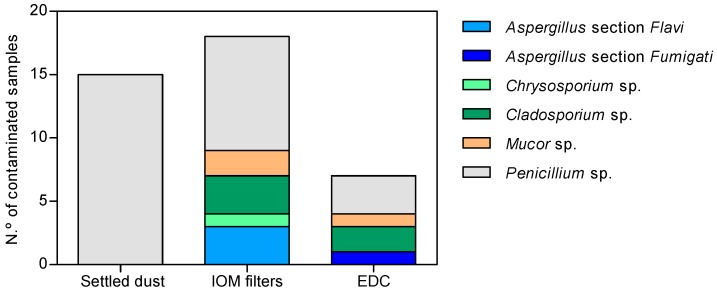
Fungal distribution in IOM filters, settled dust, and EDC samples in all media (SDA, ITRA, VORI, and POSA).

Residual growths were obtained in the azole resistance screening media at the tested concentrations. Personal exposure (IOM filters) was higher in the pizzeria restaurant, ranging from 6 CFU·m^−3^ on ITRA (including *Aspergillus* section *Flavi*) to 17 CFU·m^−3^ on VORI (Table 8), followed by Bakery 2, with 2 CFU·m^−3^ on VORI and 1 CFU·m^−3^ on ITRA (*Aspergillus* section *Flavi*) for Bakery 1. *Penicillium* sp. was only detected in the settled dust samples (Table 9), whereas a wider diversity of fungal species were detected in the EDC samples (Table 10). In Bakeries 1 and 2, the fungal load on the azole-supplemented media ranged from 106 CFU·m^−2^ (ITRA and VORI) to 1805 CFU·m^−2^ (VORI) on EDC (Table 10). In total, six fungal species were identified, the most distributed being *Penicillium* sp. (Figure 4). Of note, two species of *Aspergillus* were detected, namely: *Aspergillus* section *Flavi* in the pizzeria restaurant and Bakery 1, and *Aspergillus* section *Fumigati* in Bakery 2. Another important fungal genus detected was *Mucor* sp. in Bakery 2 (IOM filters and EDC).

### 3.5. Fungal Detection

The molecular detection, using real time PCR, for the target *Aspergillus* sections *Circumdati*, *Flavi,* and *Versicolores* was negative for all of the samples analyzed. However, *Aspergillus* section *Fumigati* was detected in one sample of settled dust (4%, 1 out of 25) and on 12 IOM samples (60%, 12 out of 20 samples). Of note, *Aspergillus* section *Fumigati* was only detected in two IOM samples from the pizzeria, similar to the results from the culture-based methods (Table 11).

### 3.6. Mycotoxins Results

**Figure 5:**
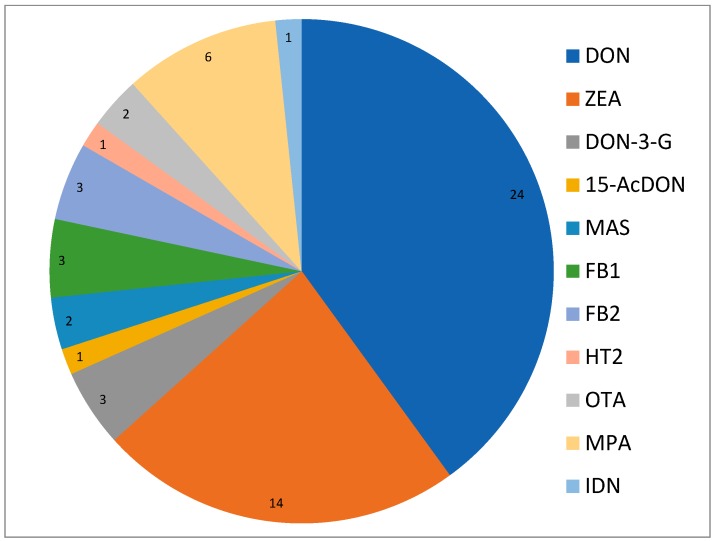
Reported frequency of mycotoxins. DON—deoxynivalenol; MAS—monoacetoxyscirpenol; OTA—ochratoxin A; MPA—mycophenolic acid; IDN—indomethacin.

DON was detected in almost all the samples (24 of 25) with values ranging between <18 and 170.1 ng/g (67.3 + 63.6). ZEA was also detected in 14 samples’ (56%) concentrations, ranging between <1.2 and 3.3 ng/g (0.8 + 0.9). DON and ZEA were the two most reported mycotoxins, however, others mycotoxins were also detected, including, DON-3-G (three samples, all <32 ng/g), 15-AcDON (one sample, <6.8 ng/g), MAS (two samples, <6.8 and 8.3 ng/g), FB1 (three samples, <4.3 and 15.61 ng/g), FB2 (three samples, all <3.7 ng/g), HT2 (one sample, 2.35 ng/g), OTA (two samples, <1.7 ng/g), MPA (six samples, values between <1.8 and 10.27 ng/g), and IDN (one sample, <0.8 ng/g; Figure 5). 

The results also showed that at least one mycotoxin was found in eight samples, two mycotoxins in six samples, and three mycotoxins were also found in six samples. Two samples were found with six and seven mycotoxins each, two samples with four mycotoxins, and only one sample had not detected mycotoxins.

### 3.7. Correlation Analysis

A significant positive correlation was found between the amount of flour dust used per day (*r*_s_ = 0.779, *p* < 0.0001) and increased exposure to flour dust, and the flour dust exposure among those working in the bakeries were significantly higher than those in the pizzeria (*p* = 0.004). A significant negative correlation was detected between the relative humidity and the total bacteria load (*r*_s_ = −0.448, *p* = 0.048), which means that the higher the relative humidity, the lower the total bacteria counted on the samples. Similarly, the fungal counts on the MEA were negatively correlated with the total Gram-negative bacteria (*r*_s_ = −0.459, *p* = 0.042). 

The fungal counts from the settled dust samples on the MEA were significantly correlated with those on DG18 (*r*_s_ = 0.420, *p* = 0.037), which means that higher fungal concentrations on MEA are related to higher concentrations on DG18. The fungal counts on the MEA were negatively correlated with the fungal counts on DG18 for the EDC samples (*r*_s_ = −0.971, *p* = 0.001), which suggests that higher fungal concentrations in the MEA on settled dust are related to lower fungal concentrations on DG18. Gram-negative bacteria were correlated with fungal counts on MEA in EDC (*r*_s_ = 0.814, *p* = 0.049), which suggests that high Gram-negative bacteria concentrations are related to high fungal concentrations on MEA (Table 12). 

As for the personal samplers, a significant correlation was detected between the fungal counts in the MEA and total bacteria counts for the EDC samples (*r*_s_ = −0.886, *p* = 0.019), which suggests that higher counts for personal samplers are related to lower total bacteria concentrations on EDC samples (Table 12).

Finally, for the EDC samples, a significant correlation was detected between the total bacteria and Gram-negative bacteria (*r*_s_ = 0.845, *p* = 0.034), which means that higher total bacteria concentrations are related to higher Gram-negative bacteria concentrations (Table 12).

Statistically significant differences were detected between the fungal counts across the three collecting units (pizzeria, Bakery 1, and Bakery 2; (χ^2^(2) = 9.778, *p* = 0.008) The fungal counts on MEA were significantly different between Bakeries 1 and 2 (*p* = 0.005), with significantly higher counts for the samples from Bakery 1. The Gram-negative bacterial counts were significantly different between the three sites (χ^2^(2) = 15,436, *p* = 0.000), where Bakery 2 differs and Bakery 1 (*p* = 0.001) and the pizzeria (*p* = 0.011), with higher levels in Bakery 2 (Table 13). No significant difference in the fungal or bacterial counts collected in the IOM filters were found between the sites.

For the EDCs, it was not possible to precede the comparison between the collection sites, as there were only two observations per site.

## 4. Discussion

In this study, we assess exposure to flour dust and its microbial constituents among workers in two bakeries and a pizzeria restaurant. To the authors’ knowledge, this is the first study to report flour dust exposures for pizzeria restaurant workers. Bakers had geometric mean exposures of 6.49 mg m^−3^, with the mean ranging from 1.29–18.29 mg m^−3^, with 90% of 8 h TWA exposures among the bakers exceeded the occupational exposure limit for flour dust (1 mg m^−3^). Inhalable dust exposure measurements for the restaurant pizzeria workers had a geometric mean of 0.87 mg m^−3^, ranging from 0.46–2.61 mg m^−3^, with 20% of 8 h TWA measurements exceeding the OEL for flour dust. 

Exposure measurements for the restaurant pizzeria are within the range of exposures reported for South African supermarket bakery supervisors and managers [24]. Bakery 2 was a much busier bakery than Bakery 1, handling significantly more (*p* < 0.05) flour per day (>100 Kg), and baking more than 800 bread and pastry products each day. The exposure concentrations in Bakery 2 are relatively high, and the arithmetic mean exposure concentrations are within the range of exposures reported for mixers and weighers in United Kingdom bakeries between 1985–2003 [13]. The concentrations in Bakery 1 are within the range of exposures reported for Norwegian bakeries [13], the measurements for bakery cleaning staff in the United Kingdom [14], and for flour mill workers and ingredient producers in the Netherlands [22]. The concentrations reported in this study suggest that bakery workers are at an increased risk for the development of flour-induced sensitization, rhinitis, and asthmas as a result of their exposure to flour dust [8,37]. To the authors’ knowledge, this is the first study in Irish bakeries and pizzerias, and so comparisons with previous Irish measurements cannot be made. However, comparisons with United Kingdom data collected between 1985 and 2003 and Norwegian exposure data for 2009–2012 suggest that this sector has high exposures.

Although a busier bakery, significantly lower fungi concentrations were detected in Bakery 2 (on MEA), but it had higher concentrations of Gram-negative bacteria compared with other sites. Different bioburden profiles were observed (fungi versus bacteria), possibly due to competition among microorganisms, and have previously been observed in similar research on occupational environments [38]. The Gram-negative bacteria and fungi contamination in indoor environments depends on several factors, such as the presence of stagnant water. Thus, a possible explanation is that the fungi and bacteria growing in water-damaged building materials could have different levels of tolerance for environmental pressures [39].

Similar to previous studies, different quantitative and qualitative fungal burden results were obtained from the passive and active sampling methodologies used (EDC and settled dust versus filtration sampling), and from the two sample media (MEA and DG18). Thus, it was possible to obtain a more complete picture regarding the microbial contamination biodiversity, justifying different sampling devices and sample media to be used in routine exposure assessments for fungi (as was the case for characterizing *Aspergillus* species) in this occupational environment [2,40,41]. Similar to the fungal characterization on MEA and DG18, azole screening also showed the presence of multiple fungal species and differences in *Aspergillus* sp. distribution across sites.

Air samples and EDC fungal counts followed the same trend than a previous study, which explored fungal concentrations in Portuguese bakeries [41]. However, regarding settled dust, in this current study, besides mycotoxin detection, fungal isolates were observed using culture-based methods and were detected by qPCR (*Aspergillus* section *Fumigati*), whereas in the Portuguese study, only mycotoxins were detected, which emphasizes the importance of measuring both fungi and mycotoxins, as the absence of one (fungi/mycotoxins) is not a surrogate for the absence of the other [42].

The presence of fast-growing fungi such as *C. sitophila* and Mucorales order (*Mucor* sp.), which are commonly found in bakeries as a result of raw materials entering the facilities [43], appeared to inhibit the growth of other fungi (with a clinical and/or toxigenic potential) on culture-based methods [2,41], and so molecular tools were used to screen for the presence of toxigenic fungal strains. *Aspergillus* section *Fumigati* was detected in a further 11 samples compared with culture-based methods, illustrating the need to use both methods in parallel.

Besides the dominance of *Penicillium* species, *Aspergillus* species were also detected, with *Aspergillus* section *Fumigati* detected in both bakeries. A previous study in 10 Portuguese bakeries (assessed by EDC and raw material samples) showed a greater fungal diversity (eleven species) and higher fungal burden (up to approximately 50,000 CFU·m^−2^ on EDC) in azole-supplemented media compared with this study, although no *Aspergillus* section *Fumigati* was found [44]. The number of *Aspergillus* sp. isolates may be underestimated in both studies because of competition with other species with faster growth rates [45] that might be present in composite environmental samples.

The emergence of azole resistance in *Aspergillus* sp., first reported as secondary resistance to itraconazole in *A. fumigatus* in 1997, is an increasing threat to human health [46,47], while it also challenges food security [48]. Exposure to *Aspergillus* section *Fumigati* is reportedly a causative agent for invasive infections in immune-compromised individuals [49,50], with most cases of azole-resistant disease originated by resistant *Aspergillus* section *Fumigati* from environmental sources [51]. Primary antifungal resistance in *Aspergillus* species is also growing, and also involves species that are common causes of invasive infections, such as *Aspergillus* section *Flavi* [46]. In our exploratory screening of susceptibility to azoles in environmental samples from bakeries and pizzerias, *Aspergillus* section *Flavi* and *Aspergillus* section *Fumigati* were found with a reduced susceptibility to azoles at the tested conditions from Bakery 1. In order to establish the clinical significance of these findings, further studies on the thermotolerance of these isolates must be conducted, as well as the reference microdilution methods so as to determine the minimal inhibitory concentration (MIC). The molecular detection of resistance mutations should also be performed so as to confirm the results of the culture-based methods.

The presence of *Mucor* sp. with a reduced susceptibility to ITRA and POSA in the assessed bakeries and pizzerias was unexpected, and should be further investigated. Mucormycosis is an increasing disease associated with a high morbidity amongst high-risk individuals [52]. Posaconazole is currently used for the treatment of mucormycosis, and itraconazole is considered effective (with species-specific activity) in vitro, whereas voriconazole lacks activity against Mucorales [53,54]. The reduced susceptibility of *Mucor* sp. from environmental samples to ITRA and POSA was contradictory to the results from a previous study on antifungal-resistant Mucorales in bakeries [55].

Data on azole-resistance for non-*A. fumigatus* fungal species (such as *Penicillium* sp. and *Cladosporium* sp.) are very limited or non-existent in environmental samples, with MIC-distributions reported worldwide, including only a limited number of clinical isolates, other than for *A.* section *Fumigati* species. In this scenario, it is difficult to distinguish in vitro susceptibility at a species level, thus, molecular identification is crucial to increase our knowledge of the susceptibility to antifungal agents. The fact that secondary resistance can emerge from environmental sources highlights the importance of the assessment of different settings outside of healthcare facilities [56,57,58,59,60].

DON was the dominant mycotoxin in terms of the frequency of the detection and magnitude, similar to findings of a previous study in a Portuguese fresh bread dough company [2], where DON was also the prominent mycotoxin in the urine samples collected from the workers, but also from the settled dust sample. This previous study concludes that workplace exposure adds significantly to the total mycotoxins’ body burden, particularly in the case of DON. Indeed, a previous report developed by Brera et.al [61], and including exposure data from three European countries (Italy, Norway, and the United Kingdom) demonstrated that intakes of pasta and pasta-like foods, breakfast cereals and snacks, and bread and bread-like foods and biscuit were significantly associated with a higher level of total DON, adjusted for creatinine. Therefore, contamination is probably coming from the cereal crops, continues in the grain farms where the grains are processed, and stored to produce various products, namely feed and flours [2,3]. Although the present study did not include a human biomonitoring element, a similar conclusion is likely here. Settled dust (composed essentially of flour) and organic dust are likely to contribute to the inhalation exposure of mycotoxins. This can happen because of the re-suspension of settled dust, and also from exposure as a result of the high volumes of flour used by the workers in this sector on a daily basis [2,3]. There is currently a knowledge gap concerning the approach, which should be used to accomplish a suitable risk assessment methodology for mycotoxins, as toxicokinetics and toxicodynamics data for mycotoxins from exposure routes other than ingestion are lacking [42]. The mycotoxin contamination of flour can vary depending on where the wheat is harvested, and is thought to be now impacted by climate change, as the cereals used for flour production can be contaminated by different mycotoxins or with a different intensity [40,41,62]. Therefore, monitoring programs for flour contamination and workers exposure should be done regularly. Additionally, the fact that most of the samples have more than one mycotoxin present (64% present more than one mycotoxin in each sample, with a maximum of seven mycotoxins present in one sample) claim attention, as in previous reports [2,40,42], that the most common exposure scenario is co-exposure to several mycotoxins. Therefore, synergistic or additive effects should also be taken into account when performing a risk assessment, and future research work developed in this type of occupational setting should look for the presence of several mycotoxins [2,42].

## 5. Conclusions

Similar to previous research work, the results suggest the potential for high exposures to organic dust and their constituents in bakeries, and also provide new data and similar exposure conclusions for pizzeria restaurants. This exposure is associated with the use of flour dust, and the fact that some of this flour dust is the perfect nutrient for fungi and bacteria to grow. Toxigenic fungal species were observed and detected by qPCR, and species with clinical relevance were observed on the azole resistance screening. The results also point to a possible exposure to mycotoxins, with flour being the probable contamination source. These findings also support previous reports where occupational exposure to mycotoxins was observed in this occupational setting. Additionally, this study showed the benefit of using a multi-approach regarding sampling methods and assays applied.

## Figures and Tables

**Table 1:** Summary characteristics of the participating bakeries and restaurant pizzeria.

Sample ID	Business Type	Flour Used	Amount of Flour Used (kg/day)	Dough Produced (pcs or kg/day)	Number of Pizza/Bakeries Produced (per day)	Work Activities or Area	Number of Samples Collected	Ventilation	Work Area (m^2^)
1.	Restaurant Pizzeria	WF; CF	18 (WF) and 0.5–1 (CF)	42 pcs	20–65 pcs	Mixing	5	Natural	6
						Preparing pizza			20
2.	Commercial Bakeries	SRF, WF, and PF	7–11 (SF), 3 (WF), and 1–2 (PF)	0	>50 pcs	General duties of bakery tasks (mixing, molding, baking, etc.)	5	Room Ventilation (Fan)	28
3.	Commercial Bakeries	T.65 (TF), (SF), and (RF)	>100 (TF), 3–9 (SP), and 3–5 (RF)	>100 kg	>800 pcs	General duties of bakery tasks (mixing, molding, baking, etc.)	5	Room ventilation (fan)	120
							5		120

WF—wheat flour; CF—corn flour; SRF—self-rising flour; PF—plain flour; TF—T.65 flour; SF—spelt flour; RF—rye flour.

**Table 2:** Limit of detection and limit of quantification for mycotoxins.

Mycotoxins	Limit of Detection (LOD; ng/g)	Limit of Quantification (LOQ; ng/g)
**Patulin**	1.1	3.6
**Nivalenol**	4.5	14.9
**Deoxynivalenol-3-glucoside**	5.4	17.8
**Deoxynivalenol**	2.7	8.9
**Fusarenon-X**	4.8	15.8
**Deepoxy-deoxynivalenol**	4.2	13.9
**α-Zearalanol**	2.0	6.6
**β-Zearalanol**	0.9	3.0
**β-Zearalenol**	1.4	4.6
**α-Zearalenol**	1.0	3.3
**Zearalanone**	0.5	1.7
**Zearalenone**	0.2	0.7
**T2 Tetraol**	5.4	17.8
**Deepoxydeoxynivalenol**	0.4	1.3
**Neosolaniol**	0.1	0.3
**15-Acetyldeoxynivalenol**	0.8	2.6
**3-Acetyldeoxynivalenol**	0.8	2.6
**Monoacetoxyscirpenol**	0.1	0.3
**Diacetoxyscirpenol**	0.3	1.0
**Aflatoxin M1**	0.1	0.3
**Aflatoxin B1**	0.1	0.3
**Aflatoxin B2**	0.1	0.3
**Aflatoxin G1**	0.1	0.3
**Aflatoxin G2**	0.1	0.3
**Fumonisin B1**	0.5	1.7
**Fumonisin B2**	0.4	1.3
**Fumonisin B3**	0.5	1.7
**T2 Triol**	0.3	1.0
**Roquefortine C**	0.2	0.7
**Griseofulvin**	0.1	0.3
**T2**	0.1	0.3
**HT2**	0.3	1.0
**Ochratoxin A**	0.1	0.3
**Ochratoxin B**	0.1	0.3
**Mycophenolic acid**	0.2	0.7

**Table 3:** Personal inhalable particulate concentrations in participating bakeries and pizzeria.

Sampling Location	*n*	Sample Duration (m)	Inhalable Dust (mg m^−3^)			
			AM	GM	GSD	Range
Pizzeria	5	300–385	1.08	0.87	1.97	0.46–2.61
Bakery 1	5	185–300	3.58	2.66	2.19	1.29–9.91
Bakery 2	10	185–250	11.1	10.14	1.57	5.82–18.29
Bakery total	15	185–300	8.59	6.49	2.35	1.29–18.29

**Table 4:** Summary of the particulate and microbial concentrations in the personal samples.

Location/Sample Number	Total Inhalable Dust (8 h TWA) (mg·m^−3^)	Fungal Isolates MEA (CFU/m^3^)	Fungal Isolates MEA (CFU/m^3^)	Fungal Isolates DG18 (CFU/m^3^)	Total Bacteria Isolates (CFU/m^3^)	Gram-Negative Bacteria (CFU/m^3^)
P01	1.05	646	0	0	0	0
P02	2.61	661	9	27	8	0
P03	0.46	656	23	34	4	0
P04	0.59	660	0	1	7	0
P05	0.70	2	2	0	10	0
B101	9.91	1201	0	0	2	0
B102	2.49	1120	0	0	11	0
B103	2.56	1179	0	0	19	0
B104	1.66	2	2	0	0	0
B105	1.29	1331	0	0	5	0
B201	13.39	0	0	0	12	0
B202	5.79	1100	0	0	2	0
B203	13.73	2	2	2	14	2
B204	7.38	0	0	0	2	0
B205	16.45	0	0	4	8	24
B206	15.46	2	2	0	4	0
B207	5.82	2	2	0	9	2
B208	8.09	1167	0	0	0	0
B209	18.29	1325	0	0	8	0
B210	6.68	1195	17	12	9	0

P—pizzeria; B1—Bakery 1; B2—Bakery 2. MEA—malt extract agar; CFU—colony-forming units.

**Table 5:** Fungal contamination distribution on IOM filters samples by units.

Sampling Location	MEA			DG18		
	ID	CFU·m^−3^	%	ID	CFU·m^−3^	%
**Pizzeria Restaurant**	*Chrysonilia sitophila*	2591.43	98.78	*Penicillium* sp.	57.71	91.77
	*Penicillium* sp.	24.22	0.92	*Aspergillus* sp.	3.87	6.16
	*Aspergillus* sp.	7.71	0.29	*Cladosporium* sp.	1.30	2.07
	Total	2623.36	100.00	Total	62.89	100.00
**Bakery 1**	*C. sitophila*	4831.13	99.95			
	*Penicillium* sp.	2.33	0.05			
	Total	4833.46	100.00			
**Bakery 2**	*C. sitophila*	4770.79	99.52	*Penicillium* sp.	15.76	88.64
	*Penicillium* sp.	18.16	0.38	*Cladosporium* sp.	2.02	11.36
	*Chrysosporium* sp.	4.71	0.10	Total	17.78	100.00
	Total	4793.66	100.00			

**Table 6:** Fungal contamination in settled dust samples by units.

Sampling Location	MEA			DG18		
	ID	CFU·g^−1^	%	ID	CFU·g^−1^	%
**Pizzeria Restaurant**	*Penicillium* sp.	13	100.00	*Penicillium* sp.	24	85.71
				*Aspergillus* sp.	2	7.14
				*Chrysosporium* sp.	1	3.57
				*Cladosporium* sp.	1	3.57
				Total	28	100.00
**Bakery 1**	*Penicillium* sp.	9	75.00	*Penicillium* sp.	30	78.95
	*Aspergillus* sp.	2	16.67	*Aspergillus* sp.	6	15.79
	*Mucor* sp.	1	8.33	*Chrysosporium* sp.	2	5.26
	Total	12	100.00	Total	38	100.00
**Bakery 2**	*Penicillium* sp.	4	57.14	*Penicillium* sp.	37	100.00
	*Aspergillus* sp.	3	42.86			
	Total	7	100.00			

**Table 7:** Fungal contamination in EDC samples by units.

Sampling Location	MEA			DG18		
	ID	CFU·m^−2^	%	ID	CFU·m^−2^	%
**Pizzeria** **Restaurant**	*Aspergillus* sp.	106.16	100.00	*Penicillium* sp.	2229.30	95.45
				*Mucor* sp.	106.16	4.55
				Total	2335.46	100.00
**Bakery 1**	*Penicillium* sp.	318.47	75.00	*Penicillium* sp.	849.26	80
	*Mucor* sp.	106.16	25.00	*Aspergillus* sp.	106.16	10
	Total	424.63	100.00	*Monascus ruber*	106.16	10
				Total	1061.57	100
**Bakery 2**	*Penicillium* sp.	4989.38	88.68	*Penicillium* sp.	2335.46	70.97
	*Chrysosporium* sp.	530.79	9.43	*Cladosporium* sp.	955.41	29.03
	*Cladosporium* sp.	106.16	1.89	Total	3290.87	100
	Total	5626.327	100.00			

**Table 8:** Fungal load in Sabouraud dextrose agar (SDA) and azole-supplemented media in IOM filter samples. ITRA—itraconazole; VORI—voriconazole; POSA—posaconazole.

Sampling Location	ID	SDA		ITRA		VORI		POSA	
		CFU·m^−3^	%	CFU·m^−3^	%	CFU·m^−3^	%	CFU·m^−3^	%
**Pizzeria** **Restaurant**	*Penicillium* sp.	16	94.12	4	66.67	17	100.00		
	*Aspergillus* section *Flavi*			1	16.67				
	*Cladosporium* sp.			1	16.67				
	*Chrysosporium* sp.	1	5.88						
	Total	17	100.00	6	100.00	17	100.00		
**Bakery 1**	*Penicillium* sp.	2	66.67						
	*Aspergillus* section *Flavi*			1	100.00				
	*Cladosporium* sp.	1	33.33						
	Total	3	100.00	1	100.00				
**Bakery 2**	*Penicillium* sp.	4	80.00			1	50.00		
	*Mucor* sp.	1	20.00			1	50.00		
	Total	5	100.00			2	100.00		

**Table 9:** Fungal load in SDA and azole-supplemented media in settled dust samples.

Sampling Location	ID	SDA		ITRA		VORI		POSA	
		CFU·g^−1^	%	CFU·g^−1^	%	CFU·g^−1^	%	CFU·g^−1^	%
**Pizzeria** **Restaurant**	*Penicillium* sp.					3	100.00		
**Bakery 1**	*Penicillium* sp.	1	100.00						
**Bakery 2**	*Penicillium* sp.	2	100.00						

**Table 10:** Fungal load in SDA and azole-supplemented media in EDC samples.

Sampling Location		SDA		ITRA		VORI		POSA	
	ID	CFU·m^−2^	%	CFU·m^−2^	%	CFU·m^−2^	%	CFU·m^−2^	%
**Pizzeria Restaurant**	n.d.								
	Total								
**Bakery 1**	*Penicillium* sp.	106.16	100.00			106.16	50.00		
	*Cladosporium* sp.					106.16	50.00		
	Total	106.16	100.00			212.31	100.00		
**Bakery 2**	*Penicillium* sp.	3609.34	94.44			1804.67	100.00		
	*Mucor* sp.	106.16	2.78	106.157	50.00			106.16	100.00
	*A.* section *Fumigati*	106.16	2.78						
	*Cladosporium* sp.			106.157	50.00				
	Total	3821.66	100.00	212.31	100.00			106.16	100.00

**Table 11:** *Aspergillus* section *Fumigati* detection results.

Sampling Location	Environmental Matrix	*C*_T_
Pizzeria	Settled dust	34.44
	IOM Filters	37.50
		37.22 *
		37.42 *
		39.50
Bakery 1	IOM Filters	36.10
		39.52
		36.34
		36.97
		38.19
Bakery 2	IOM Filters	37.95
		37.78
		33.50

* Identified by culture-based methods.

**Table 12:** Results of Spearman’s correlation analysis for the study of the relationship of the fungal concentration in the MEA, DG18, total bacteria (TSA), and Gram-negative (RB) on settled dust, personal samples, and EDC.

Samples Type	Culture Media	Settled Dust			Personal Samplers				EDC			
		DG18	TSA	RB	MEA	DG18	TSA	RB	MEA	DG18	TSA	RB
Settled dust	MEA	0.420 *	0.116	−0.345	0.021	−0.015	−0.026	−0.066	−0.642	−0.971 **	−0.588	−0.418
	DG18		0.035	−0.273	−0.029	0.271	0.075	0.187	−0.221	−0.116	0.116	0.412
	TSA			−0.125	−0.015	0.277	0.021	−0.004	−0.493	−0.152	−0.395	−0.108
	RB				−0.371	0.091	0.009	0.413	0.814 *	0.463	0.494	0.237
Personal samplers	MEA					−0.067	−0.056	−0.437	−0.145	−0.200	−0.886 *	−0.676
	DG18						0.195	0.284	−0.531	0.393	0.393	0.465
	TSA							0.329	−0.118	0.000	0.232	0.686
	RB								-	-	-	-
EDC	MEA									0.551	0.203	0.017
	DG18										0.486	0.372
	TSA											0.845*

* Correlation is significant at the 0.05 level (two-tailed); ** Correlation is significant at the 0.01 level (two-tailed).

**Table 13:** Kruskal–Wallis test results for the study of the comparison of the fungal concentration on MEA, DG18, total bacteria (TSA), and Gram-negative medium (RB) counts for settled dust, button filters, and EDC.

Culture Media		Sampling Location	*n*	Ranks	Test Statistics			Kruskal–Wallis Multiple Comparisons
				Mean Rank	Chi-Square	df	*p*	
Settled dust	MEA	Pizzeria	10	12.70	9.778	2	0.008 *	Bakery 1 ≠ Bakery 2 (*p* = 0.005)
		Bakery 1	5	21.30				
		Bakery 2	10	9.15				
		Total	25					
	DG18	Pizzeria	10	11.50	4.118	2	0.128	
		Bakery 1	5	18.70				
		Bakery 2	10	11.65				
		Total	25					
	TSA	Pizzeria	10	11.70	0.629	2	0.730	
		Bakery 1	5	13.50				
		Bakery 2	10	14.05				
		Total	25					
	RB	Pizzeria	10	10.30	15.436	2	0.000 *	Bakery 1 ≠ Bakery 2 (*p* = 0.001)
		Bakery 1	5	5.50				Pizzeria ≠ Bakery 2 (*p* = 0.011)
		Bakery 2	10	19.45				
		Total	25					
Personal samplers	MEA	Pizzeria	5	9.60	3.649	2	0.161	
		Bakery 1	5	14.80				
		Bakery 2	10	8.80				
		Total	20					
	DG18	Pizzeria	5	13.80	4.329	2	0.115	
		Bakery 1	5	7.50				
		Bakery 2	10	10.35				
		Total	20					
	TSA	Pizzeria	5	9.50	0.204	2	0.903	
		Bakery 1	5	10.60				
		Bakery 2	10	10.95				
		Total	20					
	RB	Pizzeria	5	9.00	3.333	2	0.189	
		Bakery 1	5	9.00				
		Bakery 2	10	12.00				
		Total	20					

* Statisticallly significant differences.
